# Surgeons’ prioritization of emergency abdominal surgery and its impact on postoperative outcomes

**DOI:** 10.1007/s00423-025-03723-7

**Published:** 2025-05-07

**Authors:** Severin Gloor, Antonio Wyss, Daniel Candinas, Beat Schnüriger

**Affiliations:** Department of Visceral Surgery and Medicine, Inselspital, Bern University Hospital, University of Bern, Freiburgstrasse, 3010 Bern, Switzerland

**Keywords:** Emergency abdominal surgery, Surgeons’ prioritization, Protocol violation, Postoperative outcomes

## Abstract

**Background:**

Emergency general abdominal surgery (EGS) is associated with high morbidity and mortality. Timely intervention and effective triage systems are crucial to improve outcomes. This study evaluates the impact of surgeons’ prioritization and adherence to a triage protocol on postoperative outcomes.

**Methods:**

Single-center retrospective analysis of patients undergoing EGS at Bern University Hospital from 03/2015–12/2022. Patients were categorized into four triage levels based on the urgency of surgery (level 1 within 1 h, level 2 within 6 h, level 3 within 12 h, and level 4 within 24 h). “Protocol violation” was defined in cases where the delay to surgery exceeded the triage level. Primary endpoint included complications according to Clavien-Dindo classification in patients with versus without “protocol violation”.

**Results:**

A total of 1′947 patients were included. The mean overall delay from admission to surgery was in triage level 1 69.5 ± 127.5 min., in triage level 2 206.5 ± 178.0 min., in triage level 3 350.6 ± 282.6 min. and in triage level 4 693.4 ± 354.8 min.. Triage levels 1 and 2 correlated significantly with increased complication rates compared to triage level 3 and 4 (64% vs. 43% vs. 11% vs. 10%, *p* < *0.001*). Similarly, mortality rates decreased significantly from triage level 1 through 4 (26% vs. 7% vs. 1% vs. 2%, *p* < *0.001*). “Protocol violation” occurred in a total of 13% of patients with decreasing proportions from triage level 1 to 4 (37% vs. 13% vs. 12% vs. 0%, *p* < *0.001*). “Protocol violation” did not statistically affect overall morbidity and mortality in most of the diagnoses. In patients with intestinal ischemia or abdominal abscesses, mortality was significantly higher in patients with “protocol violation”. In contrast, in patients suffering from acute inguinal hernias or gastrointestinal bleeding, morbidity was significantly higher in patients without “protocol violation”. A significantly shorter hospital length of stay (HLOS) was shown in triage level 2 and triage level 3 when patients were treated without “protocol violation” (8.6 ± 10.0 days vs. 13.5 ± 17.3 days, *p* = 0.022 and 5.3 ± 8.7 days vs. 6.4 ± 6.7 days, *p* < 0.001, respectively).

**Conclusion:**

Surgeons’ triage levels significantly correlated with mortality and morbidity. Moreover, “protocol violation” resulted in higher mortality in patients suffering from mesenteric ischemia and abdominal abscesses and resulted in prolonged HLOS. Further incorporating objective parameters into triage decisions in the EGS population may enhance prioritization accuracy, patient safety and resource utilization.

## Introduction

Surgical procedures are among the most frequent healthcare-associated interventions and remain to be associated with significant morbidity. In western countries, more than 20′000 surgical procedures are performed per 100′000 inhabitants [1]. In Switzerland, more than 1′200′000 surgical procedures are performed per year in a hospitalized or outpatient setting [2]. Significant parts of these surgical procedures need to be performed in an emergency setting due to acute diseases. At our center, during the last years approximately 30% of all abdominal surgical procedures were done in an emergency setting [3]. These emergency surgical procedures have an eight time higher mortality and result in higher health care costs than the same surgical procedures performed in an elective setting [4]. These emergencies are of different urgency depending on the underlying disease.

Early diagnosis and treatment of emergency patients is crucial to improve outcomes [5]. To schedule and prioritize the emergency surgical procedures, a triage or traffic light color system is used in many countries [5, 6]. At our institution, the surgeon on call defines the urgency and is prioritizing the intervention accordingly. However, due to reduced operating room capacities including staff (e.g. anesthesiologists, operating room technicians, nurses or surgeons), delays to surgery may occur [7].

Research regarding triage protocols in case of non-trauma emergency surgery is rare and data on its impact on postoperative outcomes are lacking. Available studies are focusing on the impact on the differences in mortality related to the delay from admission to surgical procedure [8–17]. In those studies, the prioritization or the violation from the triage decisions of emergency surgical procedures were not specifically addressed. Thus, the analysis of a large dataset of patients undergoing emergency general abdominal surgery (EGS) with a consistent triage protocol in place may answer the question whether patient’s outcomes are worse, when a violation from the protocol occurs and whether the triage decisions were adequate.

## Methods

This trial is a monocentric retrospective analysis of patients undergoing EGS at the Department of Visceral Surgery and Medicine at Bern University Hospital, University of Bern, Switzerland from March 2015 to December 2022. This study was initiated after obtaining approval from the Ethics Commission of the Canton of Bern (KEK Nr. 2023–00724).

### Patient inclusion criteria

The inclusion criteria were adult patients > 18 years of age, who underwent EGS. EGS was performed for the following conditions: acute appendicitis, acute gallbladder disease, acute bowel obstruction, acute proctologic disease, acute abdominal wall hernia (including both incarcerated and painful but reducible hernias), intestinal perforation, intestinal ischemia, abdominal abscess, colonic diverticular disease, acute inguinal hernia (also including both incarcerated and painful but reducible cases), gastrointestinal bleeding and acute pancreatitis. Surgical intervention for acute pancreatitis was limited to cases involving suspected or confirmed infected pancreatic necrosis unresponsive to interventional drainage or endoscopic therapy, or cases of abdominal compartment syndrome. There had to be a documented time of admission to the emergency department, as well as a time of registration of the operation within the electronic registration system and the time of the skin incision at the start of operation. Additionally, the documentation of the triage level as well as the Clavien-Dindo classification of complications was required for inclusion [18, 19]. Patients who underwent organ transplantation, suffered traumatic injury or were treated due to a complication after elective surgery were excluded from analysis.

### Emergency general surgery triage system

At our institution, EGS patients are prioritized using a four-level triage system. This system is based on the clinical judgment of the surgical team on call including the patient’s physiological status, the suspected underlying pathology and the urgency of surgical intervention. Each level reflects the recommended maximum time interval between decision to operate and incision. Following ample system was in place:

1. Triage Level 1 – Immediate (within 1 hour):
  This level is reserved for patients in life-threatening conditions requiring rapid surgical intervention to prevent imminent death or irreversible organ damage.
  *Examples:* Suspected intestinal ischemia with hemodynamic instability, perforated hollow viscus with generalized peritonitis and shock, uncontrolled intra-abdominal bleeding, abdominal compartment syndrome.
2. Triage Level 2 – Urgent (within 6 hours):
  Patients who are hemodynamically stable on admission, however that are likely to deteriorate if not treated within hours.
  *Examples:* Perforated hollow viscus without shock on admission, inguinal hernia with suspected bowel compromise, adhesive small bowel obstruction with peritonitis.
3. Triage Level 3 – Semi-urgent (within 12 hours):
  Patients with conditions requiring surgical intervention that are stable and not at risk of deterioration in the short term.
  *Examples:* Acute cholecystitis without signs of sepsis, acute appendicitis, localized intra-abdominal abscess without shock.
4. Triage Level 4 – Delayed (within 24 hours):
  Conditions that require surgery during the admission but do not pose an immediate threat to life or organ function.
  *Examples:* Symptomatic inguinal hernia without signs of strangulation, symptomatic cholecystolithiasis, perianal abscess.

In case of a required EGS procedure, the surgeon on call consented the patient and entered details of the planned surgical procedure and level of urgency according above mentioned triage system into an electronic registration system. At the same time, the anesthesiologist on call as well as the OR personnel (operating room technicians) were informed by phone call by the surgeons on call. A “protocol violation” was defined as an in-hospital delay to surgery that exceeded the time recommended by the patient’s triage level (e.g. a patients triaged at a level 2 waiting longer than 6 h until start of the operation).

### Primary and secondary endpoints

The study objective was to assess the impact of prioritization and “protocol violation” on outcomes of patients undergoing EGS. Primary endpoint was the occurrence of complications according to the Clavien-Dindo classification. Secondary endpoints were mortality, hospital length of stay (HLOS) and rate of “protocol violation” according triage level.

### Data extraction

At Bern University Hospital, clinical data is documented routinely in different electronic clinical data platforms. Following variables were collected from these data platforms for study purposes: demographics (age, sex, height, body weight), American Society of Anesthesiology (ASA) score, mortality, HLOS, level of urgency according above mentioned ample system, and times including admission to the ED, notification of OR personnel and start of operation. Disease characteristics, comorbidities and postoperative complications were extracted according to their International Statistical Classification of Diseases and Related Health Problems Code (ICD).

### Statistical analyses

Quantitative and qualitative variables were expressed as mean ± standard deviation or percentages. Number and severity of complications were classified according the Clavien-Dindo score [19]. Demographics, disease characteristics as well as primary and secondary endpoints were compared using Fisher exact test for categorical variables and the Mann–Whitney U test for continuous variables, as appropriate. *P* < 0.05 was considered statistically significant. Statistical analyses were performed using SPSS® version 28.0.1.1 (IBM, Armonk, New York, USA), R version 3.3.2 (R Core Team, GNU GPL v2 License) and R Studio version 1.3.959 (RStudio, Inc. GNU Affero General Public License v3, Boston, MA, 2016).

## Results

**Fig. 1:**
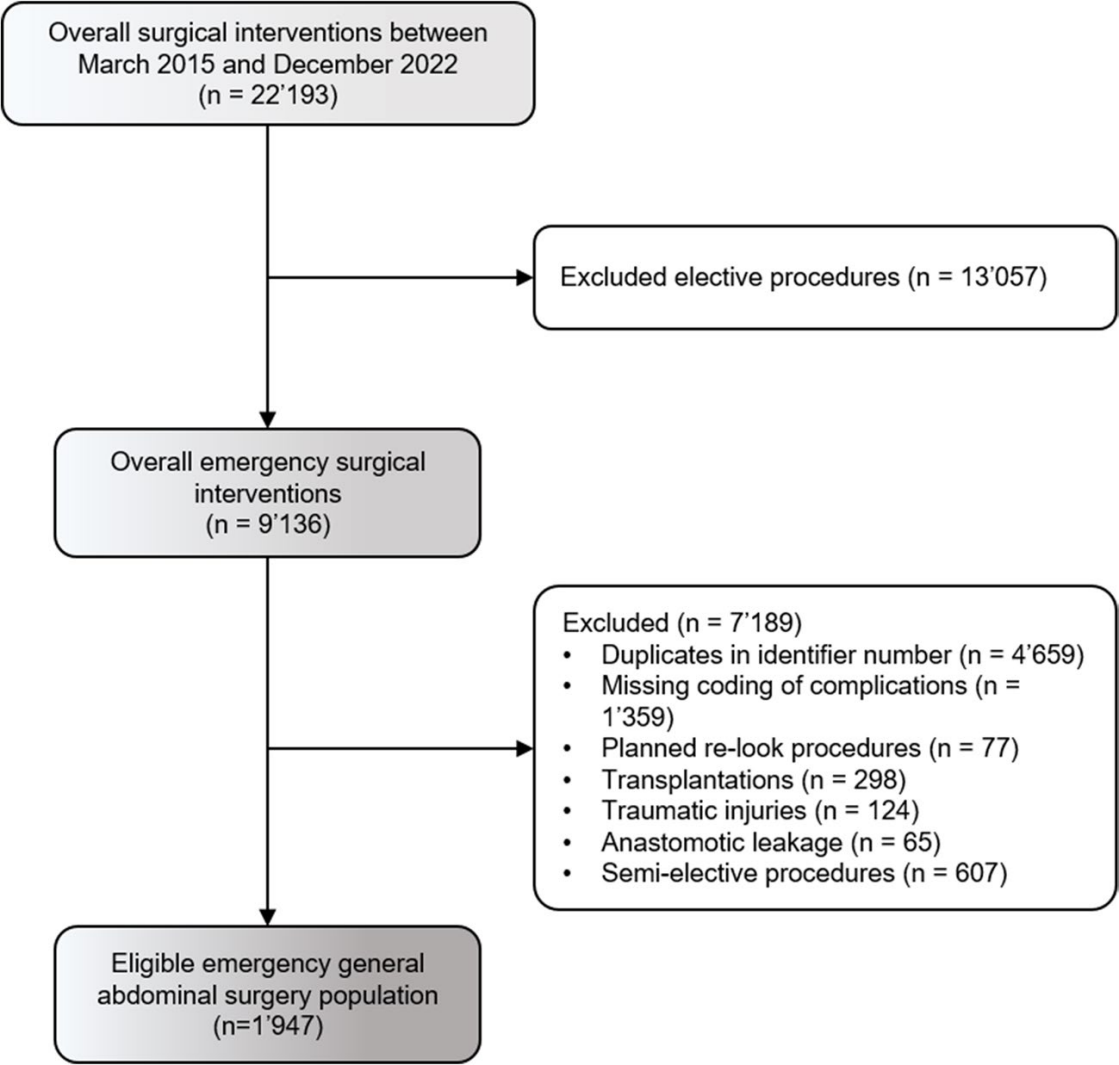
Study outline

Figure 1 shows the study outline. There were a total of 22′193 surgical interventions performed between 03/2015 and 12/2022 at the Department of Visceral Surgery and Medicine of the Bern University Hospital. After excluding elective procedures (*n* = 13′057), overall 9′136 interventions remained. After excluding 4′659 duplicates, missing coding of complication scores (*n* = 1′359), planned re-look procedures (*n* = 77), 298 transplantations, 124 trauma related operations, 65 anastomotic leakages or other semi-elective surgeries (*n* = 607) a total of 1′947 EGS patients were available for further analyses regarding primary and secondary endpoints.

### Demographics

Demographics and disease specific data of the study population are delignated in Table 1. Of note, patients in lower triage levels showed significantly higher ASA scores and more co-morbidities. Patients were treated due to the following diagnoses (Table 2): acute appendicitis (*n* = 526), acute gallbladder disease (*n* = 433), acute bowel obstruction (*n* = 256), acute proctologic disease (*n* = 167), acute abdominal wall hernia (*n* = 146), intestinal perforation (*n* = 106), intestinal ischemia (*n* = 84), abdominal abscess (*n* = 60), colonic diverticular disease (*n* = 58), acute inguinal hernia (*n* = 53), gastrointestinal bleeding (*n* = 30), or acute pancreatitis (*n* = 28). The three most common comorbidities included following organ systems: cardiovascular (*n* = 847), renal insufficiency (*n* = 462) or pulmonary disorders (*n* = 400). A total of 26% (*n* = 509) of the patients had a history of previous abdominal operation.

**Table 1:** Demographics of abdominal emergency operations stratified to triage score

Variable	Overall (*n* = 1947)	Triage Level 1 (*n* = 91)	Triage Level 2 (*n* = 932)	Triage Level 3 (*n* = 812)	Triage Level 4 (*n* = 112)	*p-*value
Age, mean years (SD)	55.0 (± 19.7)	64 (± 14.4)	61.5 (± 17.7)	46.9 (± 19.3)	52.5 (± 18.8)	< *0.001*
Age over 65 years, n (%)	732 (38)	54 (59)	466 (50)	179 (22)	33 (30)	< *0.001*
Male sex, n (%)	813 (42)	35 (39)	397 (43)	338 (42)	43 (38)	*0.752*
Body mass index, mean kg/m^2^ (SD)	27.2 (± 26.6)	28.3 (± 39.4)	28.4 (± 35.1)	25.9 (± 13.0)	28.0 (± 16.5)	*0.058*
Obesity (BMI ≥ 30 kg/m^2^), n (%)	294 (15)	12 (13)	135 (15)	124 (15)	23 (21)	*0.559*
High ASA-classification (≥ 3), n (%)	1043 (54)	84 (92)	686 (74)	228 (28)	45 (40)	< *0.001*
ASA 1, n (%)	211 (11)	0	39 (4)	159 (20)	13 (12)	
ASA 2, n (%)	680 (35)	5 (6)	203 (22)	419 (52)	53 (47)	
ASA 3, n (%)	614 (32)	17 (19)	375 (40)	190 (23)	32 (29)	
ASA 4, n (%)	369 (19)	39 (43)	281 (30)	38 (5)	11 (10)	
ASA 5, n (%)	60 (3)	28 (31)	30 (3)	0	2 (2)	
Comorbidities						
Cardiovascular disease, n (%)	847 (44)	72 (79)	522 (56)	217 (27)	36 (32)	< *0.001*
Pulmonary disease, n (%)	400 (21)	54 (59)	274 (29)	59 (7)	13 (12)	< *0.001*
Renal insufficiency, n (%)	462 (24)	49 (54)	314 (34)	76 (9)	23 (21)	< *0.001*
Diabetes, n (%)	251 (13)	24 (26)	150 (16)	64 (8)	13 (12)	< *0.001*
Psychiatric disorder, n (%)	342 (18)	36 (40)	216 (23)	76 (9)	14 (13)	< *0.001*
Malign disease, n (%)	203 (10)	17 (19)	140 (15)	41 (5)	5 (5)	< *0.001*
Previous abdominal operation, n (%)	509 (26)	45 (50)	349 (37)	93 (12)	22 (20)	< *0.001*

Percentages are calculated per column. *ASA*, American Society of Anesthesiology; *SD*, standard deviation. *P-value* was analyzed with the Kruskal–Wallis-Test

**Table 2:** Demographics of abdominal emergency operations stratified to triage score

Variable	Overall (*n* = 1947)	Triage Level 1 (*n* = 91)	Triage Level 2 (*n* = 932)	Triage Level 3 (*n* = 812)	Triage Level 4 (*n* = 112)	*p-*value
Diagnosis causing operation						
Acute appendicitis, n (%)	526	1 (< 1)	124 (24)	388 (74)	13 (2)	< *0.001*
Acute inguinal hernia, n (%)	53	1 (2)	39 (74)	9 (17)	4 (8)	< *0.001*
Intestinal ischemia, n (%)	84	23 (27)	59 (70)	0	2 (2)	< *0.001*
Acute bowel obstruction, n (%)	256	16 (6)	213 (83)	24 (9)	3 (1)	< *0.001*
Colonic diverticular disease, n (%)	58	1 (2)	50 (86)	7 (12)	0	< *0.001*
Abdominal abscess, n (%)	60	9 (15)	48 (80)	3 (5)	0	< *0.001*
Intestinal perforation, n (%)	106	8 (8)	91 (86)	4 (4)	3 (2)	< *0.001*
Acute gallbladder disease, n (%)	433	5 (1)	140 (32)	232 (54)	56 (13)	< *0.001*
Acute pancreatitis, n (%)	28	4 (14)	18 (64)	2 (7)	4 (14)	< *0.001*
Acute proctologic disease, n (%)	167	1 (1)	27 (16)	115 (69)	24 (14)	< *0.001*
Acute abdominal hernia, n (%)	146	7 (5)	111 (76)	25 (17)	3 (2)	< *0.001*
Gastrointestinal bleeding, n (%)	30	15 (50)	12 (40)	3 (10)	0	< *0.001*

Percentages of the specific diagnoses are calculated per row. *P-value* was analyzed with the Kruskal–Wallis-Test

### Primary endpoint

The outcomes of EGS patients are summarized in Table 3. The mean overall delay from notification of the OR team until skin incision was 288.2 ± 269.9 min. When stratified according the triage level, following delays were found: 69.5 ± 127.5 min in triage level 1, 206.5 ± 178.0 min in triage level 2, 350.6 ± 282.6 min in triage level 3 and 693.4 ± 354.8 min in triage level 4, respectively. The lower the triage level was, the more overall complications occurred (level 1: 64% vs. level 2: 43% vs. level 3: 11% vs. level 4: 10%, *p* < *0.001*).

**Table 3:** Primary and secondary endpoints of abdominal emergency operations stratified to triage score

Variable	Overall (*n* = 1947)	Triage Level 1 (*n* = 91)	Triage Level 2 (*n* = 932)	Triage Level 3 (*n* = 812)	Triage Level 4 (*n* = 112)	*p-*value
Overall time delay*, mean minutes (SD)	288.2 (± 269.9)	69.5 (± 127.5)	206.5 (± 178.0)	350.6 (± 282.6)	693.4 (± 354.8)	< *0.001*
Protocol violation**, n (%)	254 (13)	34 (37)	121 (13)	99 (12)	0	< *0.001*
Overall time difference to protocol***, mean minutes (SD)	−270.3 (± 288.1)	−1.5 (± 60.4)	−153.0 (± 178.4)	−369.4 (± 282.6)	−746.6 (± 354.8)	< *0.001*
Date overrun****, n (%)	420 (22)	4 (4)	143 (15)	208 (26)	65 (58)	< *0.001*
Length of stay, mean days (SD)	8.4 (± 12.7)	17.2 (± 17.2)	11.9 (± 15.2)	3.5 (± 4.6)	7.8 (± 13.2)	< *0.001*
Overall financial burden, mean CHF (SD)	9212 (± 14016)	18893 (± 18927)	13030 (± 16706)	3838 (± 5101)	8545 (± 14550)	< *0.001*
Complications (Clavien-Dindo), n (%)	565 (29)	58 (64)	404 (43)	92 (11)	11 (10)	< *0.001*
1	89 (5)	2 (2)	68 (7)	18 (2)	1 (1)	< *0.001*
2	143 (7)	8 (9)	106 (11)	27 (3)	2 (2)	< *0.001*
3a	58 (3)	1 (1)	36 (4)	20 (3)	1 (1)	*0.109*
3b	137 (7)	11 (12)	102 (11)	20 (3)	4 (4)	< *0.001*
4a	44 (2)	13 (14)	28 (3)	2 (< 1)	1 (1)	< *0.001*
4b	14 (1)	2 (2)	11 (1)	1 (< 1)	1 (1)	*0.030*
5	79 (4)	21 (23)	53 (6)	4 (1)	1 (1)	< *0.001*
High-grade complication (Clavien-Dindo ≥ 3b), n (%)	275 (14)	47 (52)	194 (21)	27 (3)	7 (6)	< *0.001*
30-day mortality, n (%)	96 (5)	24 (26)	62 (7)	8 (1)	2 (2)	< *0.001*
Time until death, mean days (SD)	567.1 (± 668.1)	265.1 (± 499.8)	560.6 (± 650.2)	765.5 (± 740.6)	689.7 (± 788.2)	< *0.001*

*CHF*, Swiss francs; *SD*, standard deviation. *Overall time delay was calculated from actual time from notification of the emergency surgical procedure until skin incision. **Protocol violation is defined as elapsed time period compared to triage score. ***Overall time difference to protocol is the correlation of skin incision to maximum allowed delay in the protocol. ****Date overrun is defined as date exceeded between notification of operation and skin incision. *P-value* was analyzed with the Kruskal–Wallis-Test

**Fig. 2:**
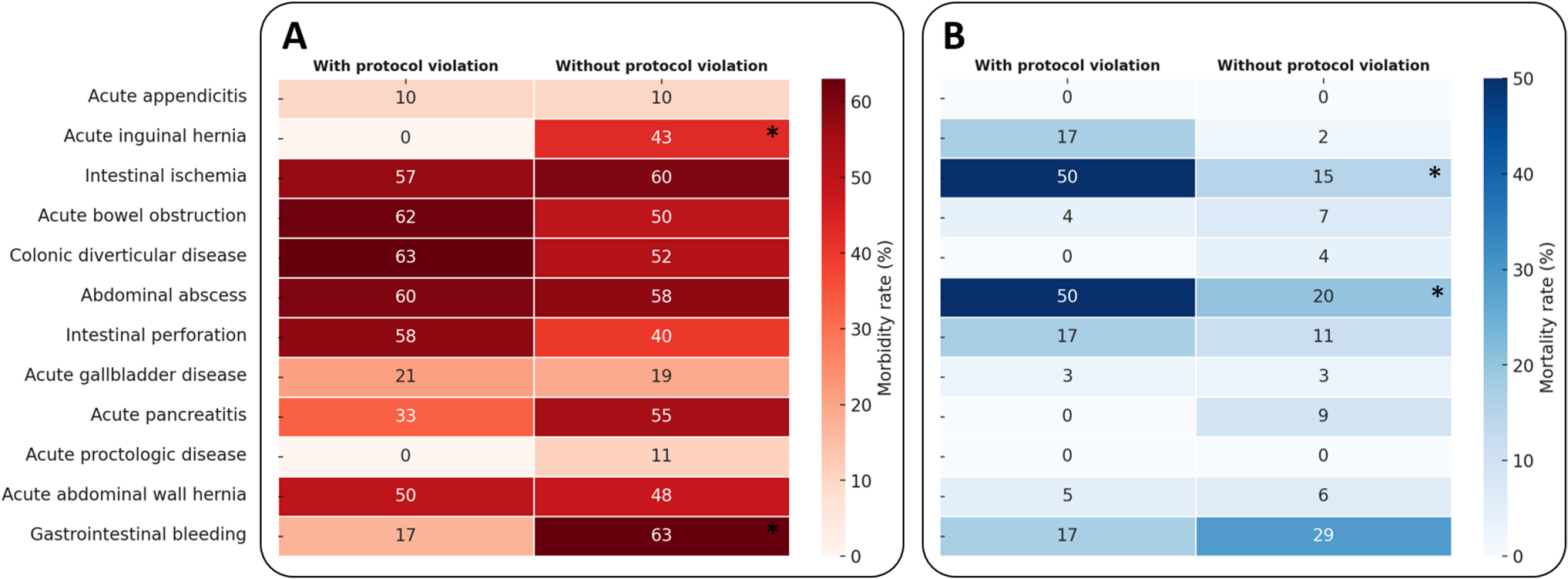
Heat-map of overall morbidity (**A**) and mortality (**B**) by diagnosis and protocol violation. * Statistically significant with *p* < *0.05*

“Protocol violation” occurred in a total of 254 (13%) patients and was significantly more frequent in lower triage level (level 1: 37% vs. level 2: 13% vs. level 3: 12% vs. level 4: 0%, *p* < *0.001*). “Protocol violation” did not statistically affect overall morbidity (Fig. 2A). Of note, in patients suffering from acute inguinal hernia or gastrointestinal bleeding, a statistically significant better outcome was observed for those with versus those without “protocol violation” (Fig. 2A).

### Secondary endpoints

Mortality rates were found to be higher in lower triage level (level 1: 26% vs. level 2: 7% vs. level 3: 1% vs. level 4: 2%, *p* < *0.001*). The highest 30-day mortality rate was found in patients undergoing EGS for gastrointestinal bleeding (27%, 8 of 30), followed by abdominal abscesses (25%, 15 of 50), intestinal ischemia (19%, 16 of 84) and intestinal perforation (11%, 12 of 106). Lower mortality was found for patients operated on for acute appendicitis (< 1%, 2 of 526), acute inguinal hernia (4%, 2 of 53), acute bowel obstruction (6%, 16 of 256), colonic diverticular disease (3%, 2 of 58), benign gallbladder disease (3%, 13 of 433), acute pancreatitis (7%, 2 of 28) or acute abdominal hernia (6%, 8 of 146). No deaths within 30 days were documented in patients operated on acute proctologic diseases. When comparing patients with or without “protocol violation” (waiting longer for surgery as triaged), intestinal ischemia and abdominal abscesses were found to have significantly increased 30-day mortality (Fig. 2B).

The HLOS was significantly longer in lower versus higher triage levels (17.2 days vs. 11.9 days vs. 3.5 days vs. 7.8 days, *p* < *0.001*). Patients in triage level 1 and 4 had a similar HLOS without “protocol violation” compared to patients with “protocol violation” (13.3 ± 13.8 vs. 13.1 ± 10.2, *p* = 0.657, and 4.5 ± 4.2 vs. 4.4 ± 3.1, *p* = 0.789). In contrast, patients with triage level 2 and 3 showed a significantly shorter HLOS, when treated without “protocol violation” (level 2: 8.6 ± 10.0 days vs. 13.5 ± 17.3 days, *p* = 0.022 and level 3: 5.3 ± 8.7 days vs. 6.4 ± 6.7 days, *p* < 0.001) A date overrun, defined as waiting over midnight until start of operation, occurred in 420 (22%) patients, and was more frequent in higher triage categories (4% vs. 15% vs. 26% vs. 58%, *p* < *0.001*) (Table 3).

## Discussion

EGS represents a significant portion of surgical procedures worldwide and remains associated with considerable morbidity and mortality. This study aimed to assess the impact of surgeons’ prioritization and adherence to a triage protocol on postoperative outcomes and HLOS. Our findings provide valuable insights into how protocol adherence can influence patient outcomes and underscore both the importance of effective triage systems in emergency surgical care as well as resource capacities to offer specific treatment.

Our analysis revealed that lower triage levels (≤ 2) were associated with significantly higher complication and mortality rates. Patients triaged at level 1 had a 64% complication rate and 26% mortality, reflecting the severity of their conditions. Conversely, patients in the higher triage levels (≥ 3) experienced fewer complications and lower mortality rates. This observation aligns with existing literature, which consistently demonstrate that the surgeons’ appreciation of the severity of a patient’s condition, including comorbidities, directly correlate with postoperative outcomes [20].

Interestingly, our study found that"protocol violations"were more frequent in the highest urgency category (level 1). However, these violations did not significantly increase overall morbidity. This finding may be explained by the fact that, in these cases, a certain degree of preoperative medical stabilization is undertaken to optimize the patient’s condition before surgery, ensuring that delays do not lead to worse outcomes, but be beneficial [21–23]. In contrast, for patients in lower urgency categories (level 2 and level 3), “protocol violations” are primarily due to organizational factors such as the unavailability of an anesthesia team or limited operating room capacity. In specific diseases such as acute inguinal hernia and gastrointestinal bleeding, patients with “protocol violations” even had better outcomes than those without delays. Importantly, there is an ongoing, implicit reassessment of all patients, ensuring that only those in stable condition experience delays. This suggests that while “protocol violations” occur, they largely affect patients who can safely tolerate the wait, reinforcing the idea that strategic prioritization plays a key role in maintaining patient safety and outcomes.

Several studies have explored the relationship between surgical delay and patient outcomes in emergency settings, as it is reported that delays beyond 24 h in performing EGS were associated with a significantly increased risk of mortality [4, 24, 25]. In a similar study, each hour of delay in source control for perforated peptic ulcers increased mortality risk by 6% [8]. Our findings support these observations, particularly in conditions like intestinal ischemia and abdominal abscesses, where delays or “protocol violation” correlated with significantly worse outcomes.

Triage systems, such as the traffic light model used at our institution, are crucial for prioritizing surgical interventions based on the urgency of diagnoses. The importance of standardized triage protocols to improve patient flow and resource allocation has been emphasized before [5]. Our study further validates this approach, demonstrating that adherence to the triage protocol correlates with improved HLOS and lower complication rates dominantly in the moderate triage levels 2 and 3. However, one of the key insights from this study is the recognition that current triage decisions, reliant on the clinical judgment and experience of the attending surgeon, may benefit from the incorporation of more objective criteria. While clinical expertise is invaluable, subjective assessments can lead to variability in triage decisions and potential delays in necessary interventions. Integrating variables (i.e. vital signs or laboratory results) into a new score, which then can assist during decision-making process, could enhance the objectivity and consistency of triage assignments, as it was made for probability calculation in sepsis or bleeding [26, 27]. This structured approach would not only support more accurate prioritization but also improve patient safety and resource utilization.

The implications of our findings for clinical practice are multifaceted. First, the data underscore the necessity of rapid surgical treatment for high-risk patients, particularly those patients with acute intestinal ischemia, where delays are detrimental. Secondly, while protocol adherence is generally beneficial, our findings suggest that a certain degree of flexibility may be warranted, allowing for individualized patient care without compromising outcomes. However, resource aspects, including operating room (OR) availability and staff shortages, remain significant challenges in adhering to triage protocols [7]. Our data revealed that date overruns were most frequent in lower urgency levels, indicating potential areas for improvement in scheduling and resource allocation.

This study’s strengths include a large number of patients and the implementation of a structured triage system applied uniformly at our institution, although the reliance on subjective clinical judgment introduces variability. However, this study has several limitations. Its retrospective design may introduce selection bias, and triage decisions were based on the subjective clinical judgment of individual surgeons without standardized scoring criteria, limiting inter-rater reliability. The analysis of additional potential confounding factors, such as surgeon experience, was not feasible due to limited availability of relevant data. Furthermore, reliance on electronic medical records introduces the risk of documentation inaccuracies. While the single-center setting ensures consistency in clinical practice, it may also restrict the generalizability of the findings to other institutions with different patient populations and resource capacities. Further research is needed to explore the nuanced relationship between surgical delay and outcomes across various emergency conditions, as there were no reasons for “protocol violation” given in particular. Prospective multicenter studies could provide broader insights and validate our findings across different healthcare settings. Additionally, evaluating the impact of targeted interventions, such as triage systems based on clinical parameters or improved OR access protocols, could inform strategies to minimize delays and enhance patient care.

## Conclusion

This study highlights the critical role of effective triage systems and timely surgical intervention in optimizing outcomes for EGS patients. While adherence to triage protocols generally improves patient outcomes, a tailored approach considering individual patient conditions and circumstances as well as resource capabilities may be warranted. Addressing operational challenges and enhancing resource allocation are essential steps in improving EGS care and reducing morbidity and mortality rates.

## Data Availability

No datasets were generated or analysed during the current study.

## Abbreviations

ASA: American Society of Anesthesiology
EGS: Emergency general abdominal surgery
ER: Emergency room
HLOS: Hospital length of stay
ICD: International Statistical Classification of Diseases and Related Health Problems
TOA: Operating room technicians
